# Reliability and performance evaluation of a solar PV-powered underground water pumping system

**DOI:** 10.1038/s41598-023-41272-5

**Published:** 2023-08-30

**Authors:** Nesma Mohamed Ahmed, Ahmed Mahrous Hassan, Mohamed Abdelwahab Kassem, Ahmed Mahmoud Hegazi, Youssef Fayez Elsaadawi

**Affiliations:** 1 Agricultural Engineering Department, Faculty of Agriculture, Cairo University, Giza, Egypt; 2 Agricultural Engineering Department, Faculty of Agriculture, Cairo University, Giza, Egypt; 3 Nuclear Research Center, Egyptian Atomic Energy Authority, Inshas, Egypt; 4 Faculty of Agricultural Engineering, Al-Azhar University, Cairo, Egypt

**Keywords:** Engineering, Solar energy

## Abstract

The operation and effectiveness of a solar-powered underground water pumping system are affected by many environmental and technical factors. The impact of these factors must be investigated to be considered when developing these systems and to ensure their dependability. This study evaluated the dependability and performance of photovoltaic water pumping system (PVWPS) under real operating conditions by examining the effects of solar irradiance, panels’ temperature, and components' efficiency. From December 2020 to June 2021, experiments were conducted on a 10 hp PVWPS located in Bani Salamah, Al-Qanater-Giza Governorate, Egypt, at latitude 30.3° N, longitude 30.8° E, and 19 m above sea level. The irradiance values reached 755.7, 792.7, and 805.7 W/m^2^ at 12:00 p.m. in December, March, and June, respectively. Furthermore, the irradiance has a significant impact on the pump flow rate, as the amount of pumped water during the day reached 129, 164.1, and 181.8 m^3^/day, respectively. The panels' temperatures rose to 35.7 °C, 39.9 °C, and 44 °C, respectively. It was observed that when the temperature rises by 1 degree Celsius, efficiency falls by 0.48%. The average efficiency of photovoltaic solar panels reached its highest value in March (13.8%) and its lowest value in December (13%).

## Introduction

The demand for electricity has increased as a result of the rapid rise in both the world's population and technology. The use of fossil fuels, which results in a significant amount of CO2 being released into the atmosphere, is one of the factors that has a significant impact on climate change. Because of these factors, many nations have begun to use a clean, accessible, renewable form of energy that is sustainable (primarily solar power)^1,2^. Diesel-powered pumps are commonly used for irrigation. However, due to an increase in the price of oil on the international market, harmful emissions from its combustion, high maintenance costs, and a short lifetime, manufacturers have been forced to find an alternative. The use of renewable energy may lessen the need for fossil fuels. Because solar energy is widely available, even in remote areas, it is a viable alternative to diesel-powered water pumps^3,4^. Solar energy is an environmentally friendly, renewable source of energy with no adverse effect on the environment when compared to fossil fuel-based sources of fuel for energy generation, and the energy can be utilized in rural areas where electricity is not easily accessible. It is one of the most important renewable energy sources that can be harnessed to generate electrical energy, which can then be used as a source of power to drive an electrical water pump for irrigation purposes^5,6^. The energy from solar radiation is primarily used to create thermal and electric energy. It is a substitute method for generating electricity for a wider range of industrial uses as well as in some other fields like building applications, food storage products, and agricultural uses to power pumps, engines, motors, and different industrial appliances like fans and refrigerators^7,8^. Using a stand-alone PV (The nomenclature are illustrated in Table 1) system in the sector of agriculture for irrigation is now becoming more popular day by day around the world. The use of solar power ensures the use of green energy in the system^9,10^. Egypt receives a lot of direct solar radiation because it is a country in the sun belt, with annual amounts ranging from 2000 to 3200 kWh/m^2^ from north to south. The sun’s shine duration ranges from 9 to 11 h, with a few cloudy days all over the year^11,12^. Solar-powered pumping systems provide water for a variety of uses, including domestic use and to fulfill the demand of water in the field of irrigation, livestock watering, and village water supply^10,13^. A PV energy generator, power converters, an electric motor, and a pump are the components of a solar-powered water pumping system^14,15^. Solar energy can be used thermally by using solar thermal collectors for heating and drying, or photovoltaically by converting sunlight into electricity using solar cells made of semiconductor materials such as silicon. Solar panels, also known as photovoltaic panels, are made by connecting solar cells in series. Both types have numerous applications in agricultural settings, making life easier and contributing to increased productivity. The solar-generated electricity can then be used to power the water pump or stored by pumping water into a high tank during the day and distributing it by gravity after dark. A battery will be required to store the energy generated during the day for electrical applications at night^16,17^. There are two methods for pumping water with a photovoltaic system: Solar energy is consumed in “real time” in the first technique, which is known as “pumping in the sun.” This solution necessitates water storage in a tank (water pumped during the day is stored for later use in the evening, for example). The second technique is to use batteries to store energy. The energy stored during the day can be used to pump water later^18^. The output power of a photovoltaic system is affected by a number of factors, including solar radiation, PV surface temperature, shadow, tilt angle, and dust accumulation. A PV system’s design should consider a number of factors and environmental conditions, including but not limited to tilt angle, irradiation, and Temperature. These variables have a significant impact on the PV’s output power^19–21^. When the solar panel surface temperature increases by 1 °C in summer and winter, the efficiency decreases by 0.48% and 0.42%, respectively^22,23^. A photovoltaic system's output power is affected by a number of factors, including PV surface temperature, tilt angle, and system component efficiencies. These factors should be researched and considered when designing and operating a PV system. When the surface of the PV module is directly perpendicular to the sun's rays, the maximum output energy from a PV cell is obtained. Because tracker orientation is oriented to maximum irradiation, it produces more PV power than horizontal orientation^24,25^. It was noticed that many of the PV water pumping stations, although well designed from an engineering point of view, face problems after that during the operation process, and also that the quantities of water pumped from the station are lower than expected. This is due to the lack of attention paid to the environmental and technical factors that have a negative impact on the station and its performance. Therefore, The aim of this work was to study the reliability and performance of the PV-powered underground water pumping system under actual operating conditions, investigate negative impact factors on the PV system, and demonstrate the possibility of relying on this system as a safe and reliable alternative to the traditional energy systems that are expensive and pollute the environment.

**Table 1:** Table of nomenclature.

Abbreviation	Description	Abbreviation	Description
PV	Photovoltaic	Isc	Short circuit Current, A
TDH	Total dynamic head, m	STC	Standard test conditions
Q	Pump flow rate, m3/hr	PDC	Power of direct current
Hf	Friction losses, m	PAC	Power of alternative current
Hst	Static head, m	Hz	Hertz
Hd	Drawdown head, m	IR	Intensity of solar radiation
Hp	Pressure head, m	Voc	Open circuit Voltage, V
HP	Hydraulic power, W	ηinv	Efficiency of the inverter
MPPT	Maximum power point tracking	ηpump	Efficiency of the pumping unit
Pmax	Maximum Power, W	ηpanels	Efficiency of the solar panels
Vmp	Maximum Power Voltage, V	ηall	Overall system efficiency
Imp	Maximum Power Current, A	DC	Direct current

## Materials and methods

Experiments were carried out in Bani Salamah, Al-Qanater, Giza Governorate, Egypt, located at latitude 30.325364° N, longitude 30.805797° E, and 19 m above sea level, from September 2020 to June 2021, and measurements were taken every 15 min through the day between sunrise and sunset.

### PV water pumping system sizing

The design of the solar water pumping system goes through several stages, and some information such as daily water consumption, static water level, and the pumping pipes length and diameter must be known. In the present case, average water consumption = 175 m^3^/day, static level = 47 m, draw down = 5 m, the pumping pipes length = 70 m, the pressure of irrigation network = 1 bar, and the pumping pipes diameter = 3 Inches = 76.2 mm.

### Pump selection

Total dynamic head TDH (m) and flow rate Q (m^3^/hr.) should be specified accurately to select the suitable pump.

### Total dynamic head TDH (m)

The friction head H_f_ (m) represents the loss of pressure in pipe due to fraction. The friction head could be calculated from Hazen William as Eq. (1)^26^.

1 $${\mathrm{H}}_{\mathrm{f}}=K\times L\times {(\frac{Q}{C})}^{1.852}\times {d}^{-4.87}$$

where H_f_ = friction losses (m) , K = constant coefficient = 1.22*10^10^, L = Length of pumping pipes (m) , Q = discharge (lit/s) , d = internal diameter of pumping pipes (mm).

$${h}_{f}={1.22*10}^{10}\times 70\times ({\frac{6.94}{150})}^{1.852}\times {76.2}^{-4.87}=3m$$

The total dynamic head TDH could be expressed as Eq. (2)^27^:

2 $${\mathrm{TDH }} = {\mathrm{ H}}_{{{\mathbf{st}}}} + {\mathrm{ H}}_{{\mathrm{d}}} + {\mathrm{H}}_{{\mathrm{f}}} + {\mathrm{H}}_{{\mathrm{p}}}$$

where H_st_ = static head (m), H_d_ = drawdown head (m), H_f_ = friction head (m), and H_p_ = Pressure head (m).

$${\mathrm{TDH}} = {47} + {5} + {3} + {1}0.{2} = {65}.{2}\;{\mathrm{m}}$$

The appropriate pump must be chosen from the Pump efficiency schemes using discharge (25 m^3^/h) and TDH (70 m). Schemes recommended a pump with 10 hp and 8 stages.

The required hydraulic power HP (W) could be expressed as Eq. (3)^22^.

3 $$HP=\frac{Q\times \rho \times g\times TH}{3600}$$

where HP = hydraulic power (W), Q = discharge (m^3^/h), ρ = water density (1000 kg/m^3^), g = Gravity acceleration (9.81 m/s^2^).

Inverter: The appropriate inverter for the pump can be chosen as follows^28^: Inverter power ≥ motor power.

$${\mathrm{Inverter}}\;{\mathrm{power}} \ge {\mathrm{motor}}\,{\mathrm{power}}.$$

Lorentz solar inverter 15 kw will be used. From inverter data sheet (MPPT voltage 500 to 600 V).

### Sizing of PV panels

The panels output drops during the morning, cloudy, and sunset periods. The total power needed to operate the pump Multiply by 1.25 determines the size of the PV panels^29^. Solar panel’s power = 1.25 × 10 hp = 12.5 hp = 12.5 hp × 745.7 W = 9321 W. Panels number = 9321/260≃36 panels.

$$\begin{aligned} {\mathrm{Solar}}\;{\mathrm{panel}}{\hbox{'}}{\mathrm{s}}\;{\mathrm{power}} = & 1.25\; \times \;{\mathrm{pump}}\;{\mathrm{motor}}\;{\mathrm{power}} \\ = & 1.25 \times 10\;{\mathrm{hp}} = 12.5\;{\mathrm{hp}} = 12.5\;{\mathrm{hp}} \times 745.7\;{\mathrm{W}} = 9321\;{\mathrm{Watts}} \\ \end{aligned}$$

$${\mathrm{Panels}}\;{\mathrm{number}} = {\mathrm{The}}\;{\mathrm{overall}}\;{\mathrm{Panels}}\;{\mathrm{power}}/{\mathrm{The}}\;{\mathrm{power}}\;{\mathrm{of}}\;{\mathrm{one}}\;{\mathrm{panel}} = {{9321}}\;{\mathrm{w}}/260\;{\mathrm{W}} = 35.{{85}}\;{\mathrm{panels}} \approx {{36}}\;{\mathrm{panels}}$$

The type of connection between panels (parallel or series) depends on the voltage and current that the inverter needs to work efficiently. As a result, according to the Lorentz inverter datasheet, the MPPT voltage range is 500 to 600 V. Therefore, every 18 panels were connected in series to form two arrays. Voltage of each array = 18 × 30.5 = 549 V.

$$\mathrm{Voltage\, of\, each\, array}=18 \times 30.5= 549\mathrm{ V}.$$

**Figure 1:**
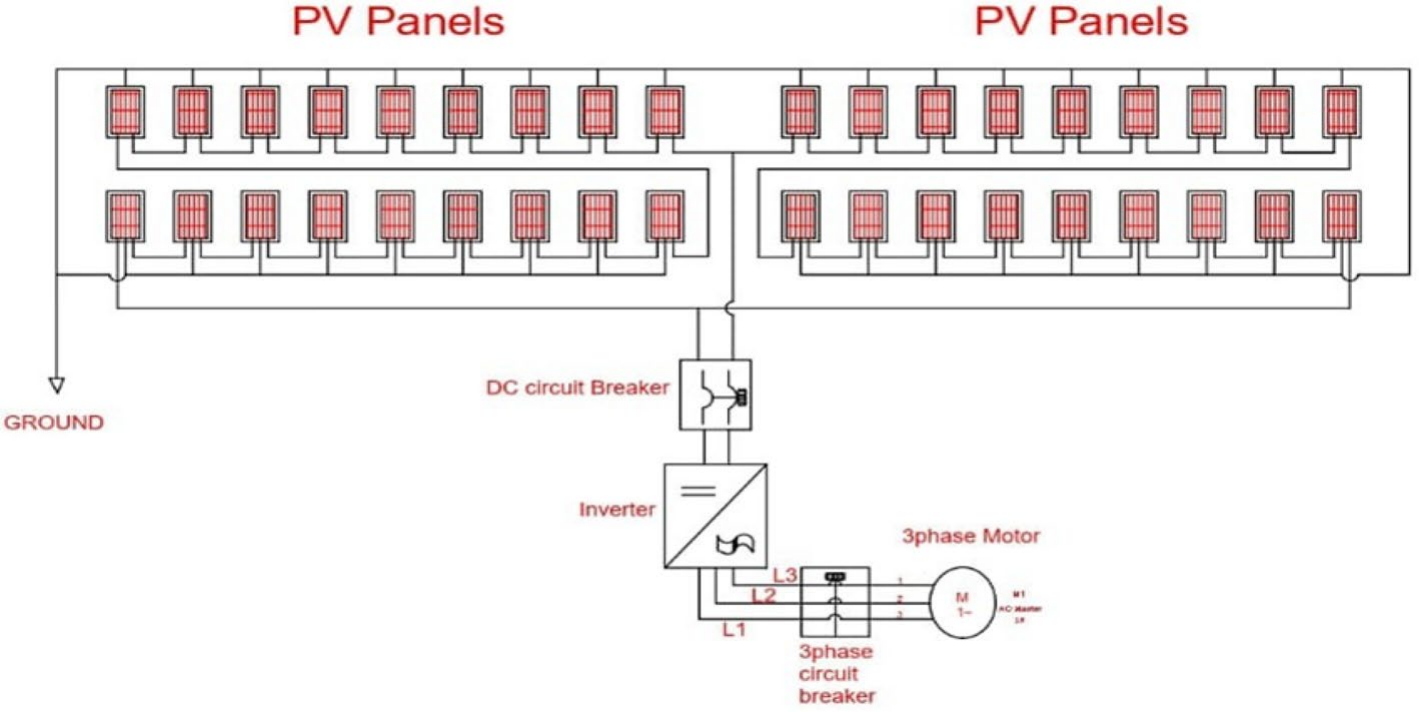
The electric diagram for PV system.

The two sets of arrays were connected in parallel in order to give a current = 2 × 8.53 = 17.06 amps. Figure 1 shows the electric diagram for a PV water pumping system, the electrical components, and connection methods.

### System installation and components

**Figure 2:**
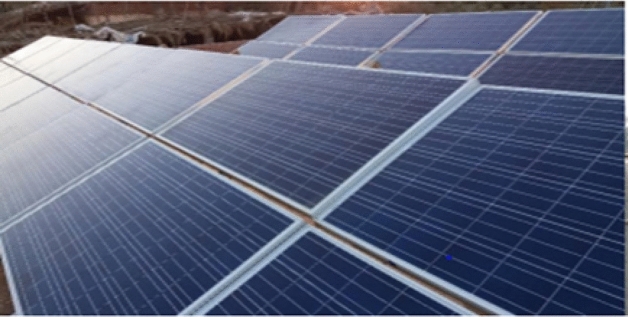
The two sets of PV arrays.

PV cells are the fundamental building blocks of almost all PV modules. To increase the voltage, panels are connected in series. Several of these strings of cells can be connected in parallel to increase current. Implemented photovoltaic system (PV) consisting of two array groupings, each of which is made up of 18 modules connected to a metal structure in series whose tilt angle can be changed manually as shown in Fig. 2. To give the inverter a current of 17 A and 549 V, two groups were linked in parallel. The type of module used in these experiments is Renesola (JC260M-24/Bb) 260 W. The datasheet for the module is illustrated in Table 2.

**Table 2:** The datasheet for the PV module.

Module Type	Renesola (JC260M-24/Bb)
Maximum Power (Pmax)	260 W
Maximum Power Voltage (Vmp)	30.5 V
Maximum Power Current (Imp)	8.53 A
Open circuit Voltage (Voc)	37.6 V
Short circuit Current (Isc)	8.95 A
Module Efficiency STC (%)	15.89%
Maximum system voltage	1000VDC (IEC)
Maximum series fuse rating	15 A

### Inverter

The inverter converts the DC power produced by the PV modules to the AC power used to drive the pump motor. It also adjusts the output frequency in real time based on the prevailing irradiation levels, and it works with MPPT (Maximum Power Point Tracking) technology to maximize power output at all irradiation levels. Table 3 illustrates the Lorentz inverter data sheet.

**Table 3:** The inverter data sheet.

Maximum output power	15 (KW)
Output voltage	380 (V)
Maximum output current	30 (A)
Maximum input power	21 (KW)
Maximum input voltage	800 (VDC)
MPPT voltage	500–600 (VDC)
Frequency	0–60 (Hz)

**Figure 3:**
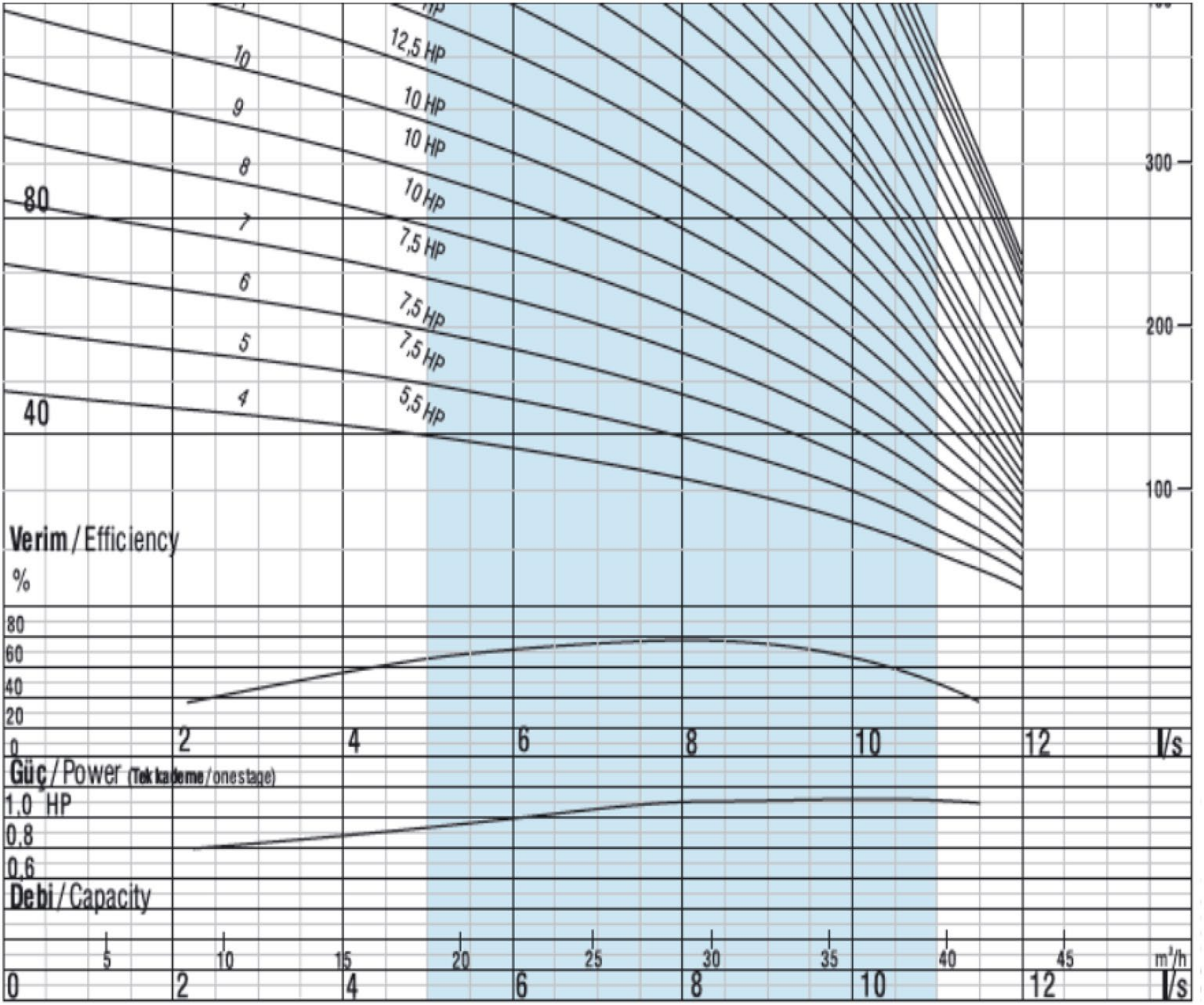
The pump performance curves.

*The pumping unit* is made up of three key components: a three-phase alternating current motor, a multistage submersible pump, and a deep well. Table 4 shows the technical data about the Vansan VSM 6/10 submersible 3-phase electric motor. The Vansan VSP-SS 06030/08 centrifugal submersible pump technical data is presented in Table 5, and the performance curves are shown in Fig. 3.

**Table 4:** The motor technical data.

Type	VSM 6/10
Power- kW	7.5
Volt- V	380
Current- A	17
Frequency-HZ	50
Efficiency	78%
Dimensions-mm	Φ = 142 L = 748
Cos φ	0.8
Revolution- rpm	2750

**Table 5:** The pump technical data.

Type	Stages	Flowrate	weight	Revolution	Dimensions (mm)		
Vsp ss 06030/08	8	30 m^3^/h	19 kg	2900 rpm	L	Φ	Shaft Φ
					1171	132	22

### Solar radiation

**Figure 4:**
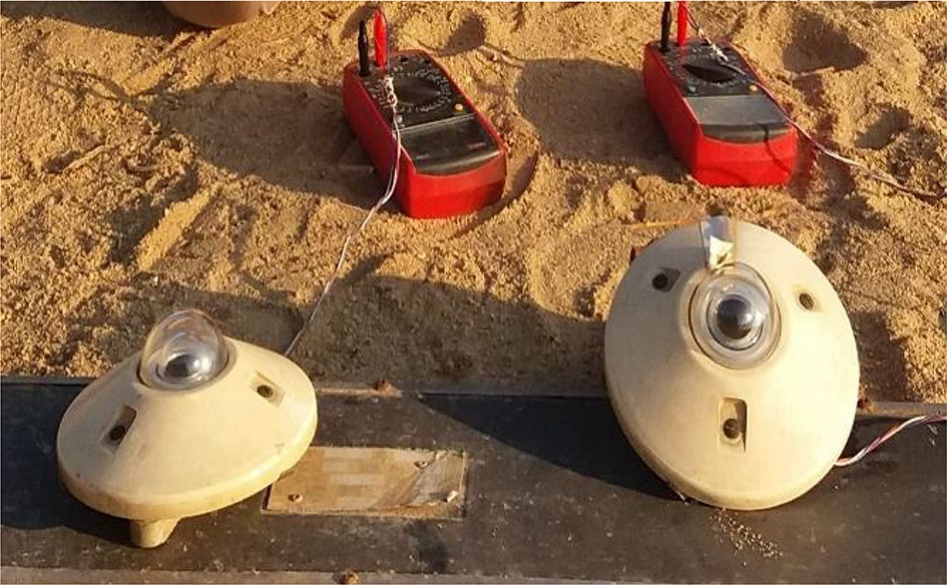
Pyranometers.

A pyranometer was used to measure solar radiation, as shown in Fig. 4. It is made up of a glass dome, a thermopile sensor, and instrument housing. Across a wide wavelength range, incoming radiation is virtually totally absorbed by a blackened horizontal surface. According to the temperature difference between the black absorbing surface and the instrument enclosure, the detector produces a very small voltage. This is on the order of 10 microvolts per square meter (W). The calibration process determines the specific sensitivity of each pyranometer, which is used to translate the output signal in microvolts into the total irradiance in W/m^2^. The sensitivity of the used KIPP&ZONEN pyranometers is (12.11*10^–6^) V/Wm^-2^ and (14.11*10^–6^) V/Wm^−2^. To convert the output signal of pyranometer in mV into global irradiance in W/m^2^ the Eq. (4) was used.

4 $${\mathrm{I}}_{{\mathrm{R}}} = \frac{mV}{{1000 \times {\mathrm{ pyranometer\,sensitivity}}}}$$

where I_R_: insolation, W/m^2^, Pyranometer sensitivity: (12.11*10^–6^) V/Wm^-2^, and (14.11*10^–6^) V/Wm^-2^, mV= Pyranometer output.

### PV Panels temperature

The solar panels’ temperature was measured every hour from sunrise to sunset with a digital infrared thermometer, and Table 6 illustrates the infrared thermometer datasheet. Also, a thermocouple thermometer was used to measure temperature, and Table 7 illustrates the infrared thermometer datasheet.

**Table 6:** Infrared thermometer data sheet.

Type	DT8011T
Temperature Range	− 50 ~ 1100 °C
Accuracy	± 2% or 2 °C
Distance to Spot Ratio	12:1
Emissivity	0.95 fixed
Response Time	≤ 0.8 s
Resolution	0.1 °C

**Table 7:** Thermocouple data sheet.

Type	k-type
Thermocouple alloy	NiCr/NiAl
Accuracy	± 0.4%
Range	− 50 ~ 750 °C

### PV system current and voltage

A UNI-T UT39C multimeter was used to measure the PV system’s output voltage and current. A multimeter, commonly referred to as a Volt/Ohm meter, is an electronic measurement device that incorporates multiple features into a single device. Voltage, current, and resistance measurements are among the capabilities of a typical multimeter. The digital multimeter’s datasheet is displayed in Table 8. Ohm's law was used to determine the power (Eq. 5).

5 $${\mathrm{P}}_{{{\mathrm{DC}}}} = {\mathrm{I}}_{{{\mathrm{DC}}}} \times {\mathrm{V}}_{{{\mathrm{DC}}}}$$

where P_DC_: PV system output power, W; I_DC_: current, ampere; V_DC_: voltage, volt.

**Table 8:** Data sheet for the digital multimeter.

Quantity	Measuring range
Model	UNI-T UT39C
DC voltage	1 mV to 1000 V
AC voltage	1 mV to 750 V
DC current	1 mA to 20A
AC current	1 mA to 20A
Resistance	1 Ohm to 20 M Ohm
Capacitance	1nF to 20 μF
Temperature	− 40 to + 1000 °C
Frequency	1 Hz to 20 kHz

### Flow meter

The 4-inch, 10-bar flow meter (ISO 4064 class B) is a device used to continuously measure, record, and display the volume of water passing through the measurement transducer under metering conditions. The flowmeter datasheet is illustrated in Table 9.

**Table 9:** The flowmeter datasheet.

Meter Size DN (mm)	100
Class	B
Q_max_–maximum flowrate m^3^/h	300
Q_n_–Nominal Flow m^3^/h	60
Q_min_–Min Flow m^3^/h	0.8
Q_t_–Transitional Flow Rate	1.8
Starting Flow m^3^/h	0.3
Min. Reading m^3^	0.01
Max. Reading m^3^	9,999,999
Accuracy between Qmin to Qt	± 5%
Accuracy between Qt to Qmax	± 2%

## Results and discussion

### The intensity of solar radiation

**Figure 5:**
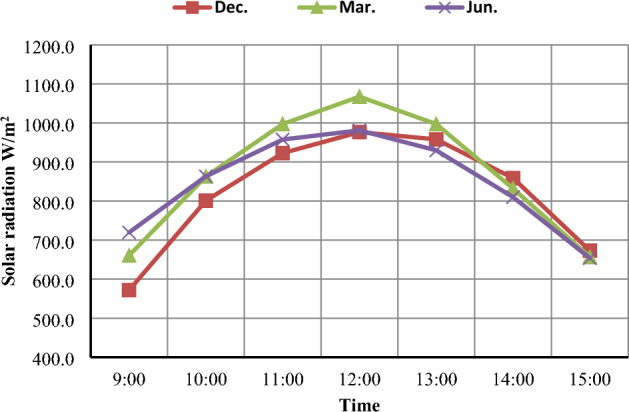
Hourly-average solar radiation in different months (December, March, and June).

The average daily sunshine hours across Egypt are about 9–11 h, so Egypt receives abundant solar energy with an annual direct solar radiation of about 2,000–3,200 kWh/m^2^/year^30^. Measurements of the intensity of the solar radiation were made using a pyranometer and digital solar radiation meter. Figure 5 shows hourly-average solar radiation (W/m^2^) for the months of December 2020, March 2021, and June 2021. Results showed that the highest values for solar radiation reached 976.5, 1067.3, and 981.0 W/m^2^, respectively, at 12:00 p.m.

**Figure 6:**
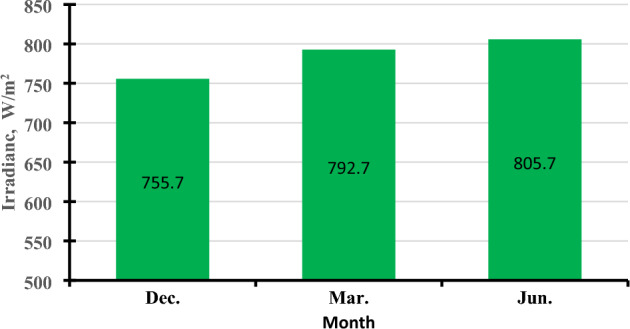
Daily-average solar radiation (W/m^2^) in different months (December, March, and June).

Daily average solar radiation is shown in Fig. 6, which illustrates the increase in daily average solar radiation in June (805.7 W/m^2^) compared to March (792.7 W/m^2^), and December (755.7 W/m^2^). It is noticeable that the intensity of solar radiation increases on sunny days and decreases on cloudy days, where clouds disperse the sun's rays. Also, the intensity of solar radiation varies with the earth`s circulation around its orbit and around the sun, where the radiation decreases in the early morning and winter (Dec.) because the altitude angle of the sun is small and the radiation penetrates a long distance of the atmosphere, while in the noon and summer (June) the intensity of the solar radiation increases because the altitude angle becomes large. and the radiation is penetrating the atmosphere over a short distance^31^.

### PV panels output current (Dc)

**Figure 7:**
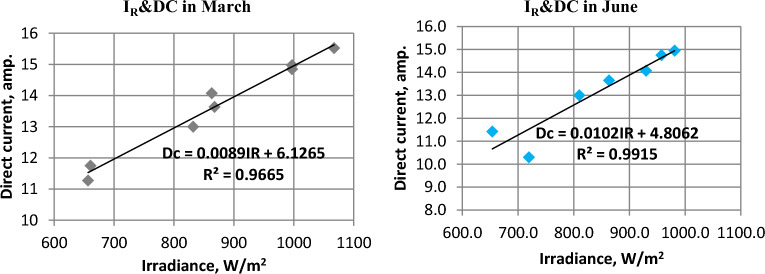
Solar radiation (I_R_) and Direct current (DC) in March and June.

**Figure 8:**
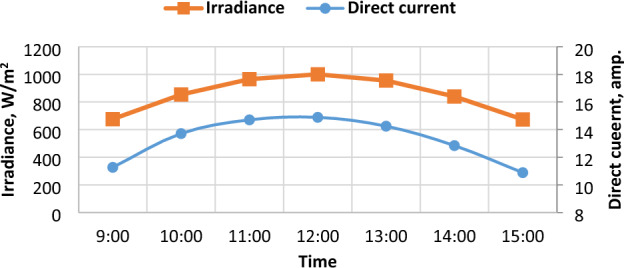
The Hourly-average radiation and DC current.

The current produced by the panels is directly and uniformly affected by solar radiation^32^. Where the produced current increases when radiation increases and decreases when solar radiation decreases. Figure 7 illustrates the correlation between direct current (DC) generated by solar panels and the intensity of solar radiation in March and June, respectively. The direct current (DC) produced by solar panels is positively affected by intensity of solar radiation as shown in Fig. 8.

### PV panels output voltage (V_DC_)

**Figure 9:**
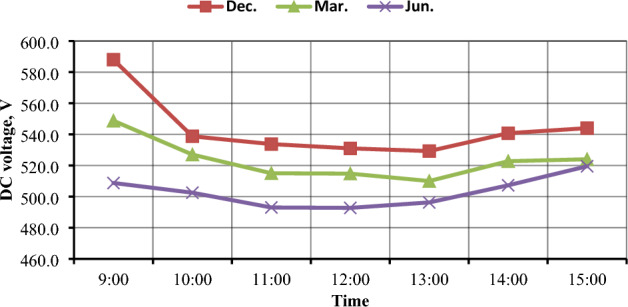
Hourly-average PV system voltage in different three months (December, March, and June).

**Figure 10:**
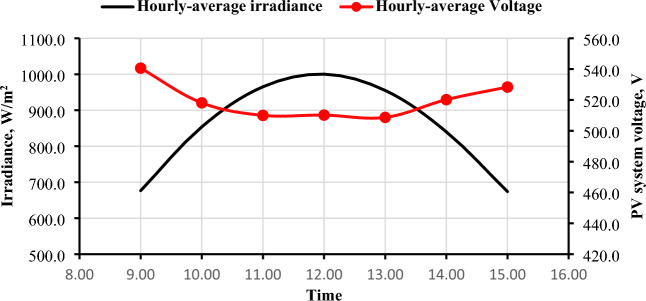
The Hourly-average radiation and PV system voltage.

The hourly average voltages that were delivered by the PV generator are shown in Fig. 9 for the months of December, March, and June. It is clear that December has the highest voltage values of all months, followed by March, and the lowest values in June. It is observed that the highest months in solar radiation and temperature were the ones with the lowest output voltage from PV systems, which may be affected by high temperatures in the summer and a clear atmosphere. It is also apparent that the voltage is not significantly affected by solar radiation^33^ as illustrated in Fig. 10.

### PV panels power (P_DC_)

**Figure 11:**
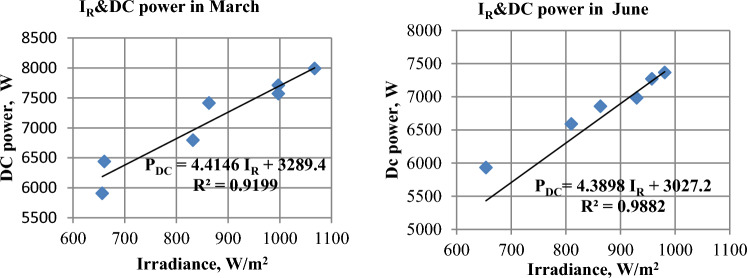
The correlation between irradiance and DC power in March and June.

**Figure 12:**
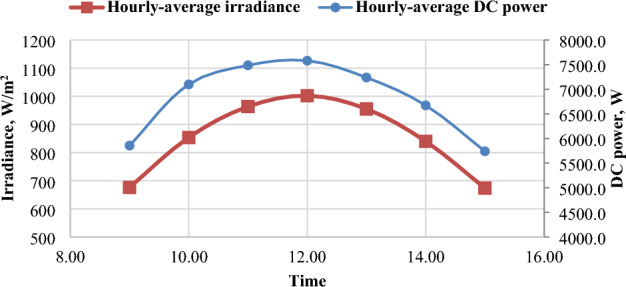
The Hourly-average radiation and DC power.

**Figure 13:**
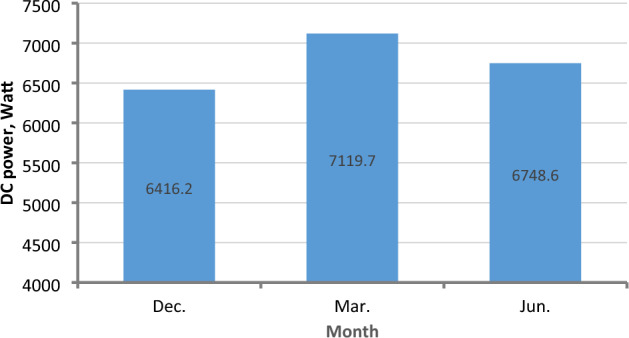
The daily-average electric Dc power at different months.

The DC power produced by solar panels is affected by the intensity of solar radiation. Figure 11 illustrates the correlation between DC power and irradiance in March and June, respectively. Figure 12 depicts the positive relationship between solar radiation and the electrical power generated by the panels, which is based on the positive relationship between radiation and electrical current. Figure 13 displays the daily-average electric DC power generated by PV panels for the months of December, March, and June. It is observed that March has the highest power values all day, followed by June, and the lowest values are in December. It’s clear that June has the highest month of solar radiation, but in this month the power was less than March because the module temperature in June was higher than March, so the efficiency in March was greater than June^33^.

### Hydraulic power that is delivered by the pump (H.P.)

**Figure 14:**
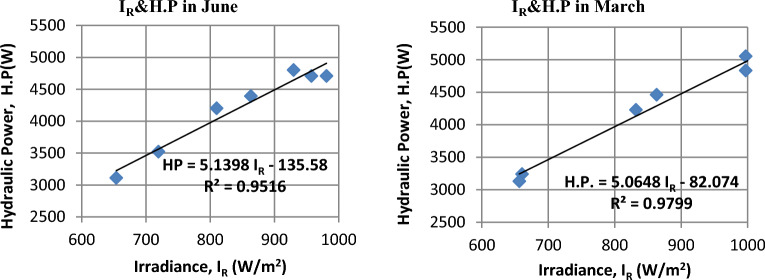
The correlation between Irradiance (I_R_) and hydraulic power (H.P) in March and June.

**Figure 15:**
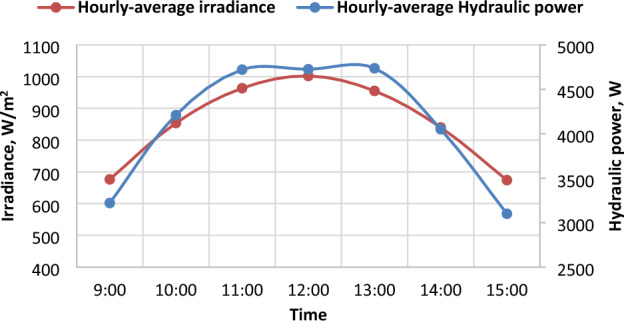
The Hourly-average radiation and hydraulic power.

It turns out there is a direct correlation between hydraulic power and the intensity of solar radiation, as shown in Fig. 14 for March and June. Experiments have revealed an increase in hydraulic power as the intensity of solar radiation increases. Figure 15 illustrates the positive relationship between solar radiation and electric power. The daily average values of hydraulic power in December, March, and June reached 3795.2, 4312.3, and 4207.4 W, respectively.

### Pump flow rate (discharge m^3^/hr)

**Figure 16:**
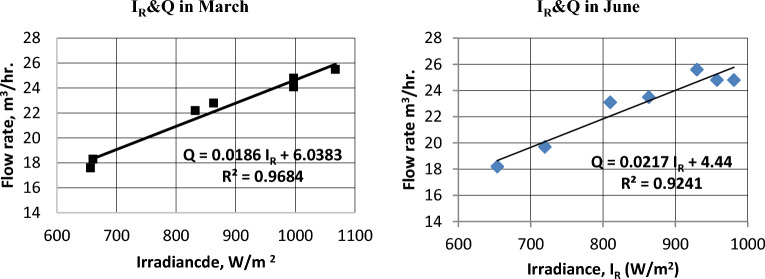
The correlation between irradiance and discharge in March and June.

**Figure 17:**
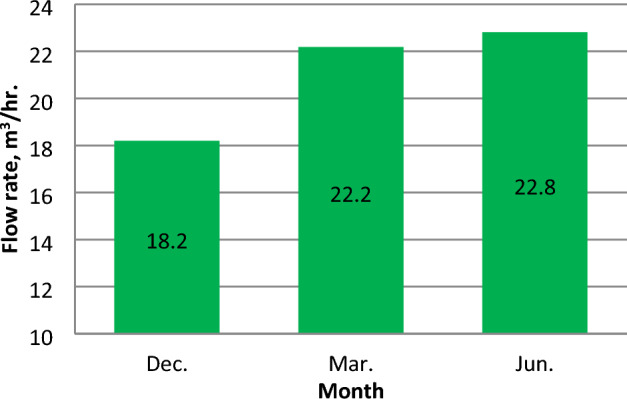
Hourly average Discharge (m^3^/hr.) at different months .

The intensity of solar radiation (I_R_) has a significant impact on pump discharge (Q)^34^. The correlation between flow rate and intensity of solar radiation is illustrated in Fig. 16 for March and June. Figure 17 shows the hourly average pump discharge through three months. The hourly average flow rate in December, March, and June reached values of 18.2, 22.2, and 22.8 m^3^/h. The number of operating hours of the pump were 7, 7, and 8 h, and the amount of water that was pumped during the day was 129, 164.1, and 181.8 m^3^/day, respectively.

### Temperature of PV panels at different seasons

**Figure 18:**
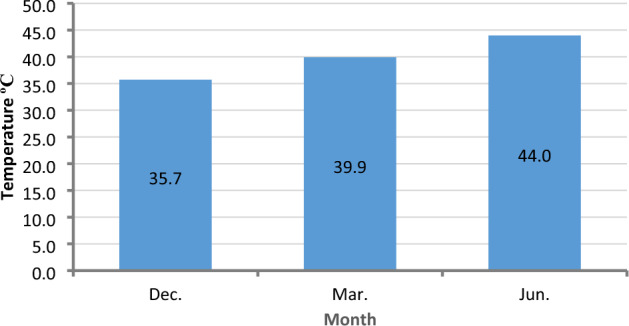
Average temperature of panels over four months.

Environmental factors surrounding solar panels directly affect solar panel production, with temperature having the greatest impact on panel efficiency^35^. Where the panel heats up and the performance of the panel degrades as a result of the increased air temperature. Figure 18 shows the average temperature of the panels with values 35.7 °C, 39.9 °C, and 44 °C in December, March, and June, respectively.

### Effect of PV panels temperature on its efficiency

**Figure 19:**
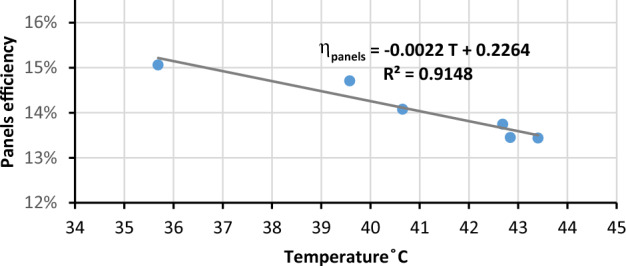
The correlation between panels temperature and its efficiency.

**Figure 20:**
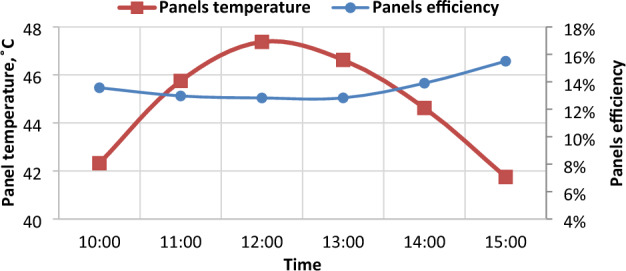
Panels temperature and panels efficiency in summer.

The efficiency of solar panels is negatively affected by temperature increasing as shown in Fig. 19. So, the performance in a high-temperature month such as June is lower than the performance in a moderate-temperature month such as March. The efficiency of panels has the lowest value at 12:00 pm because at noon the temperature has the highest value through the daytime. It’s noticeable from Fig. 20 that when temperature increases, the panels efficiency decreases, and when the temperature reaches the highest value during the day 47.4 °C at noon, the panels efficiency decreased to the lowest value 12.8%. also, it’s clear that when the temperature increases by 1 °C the panels efficiency η_panels_ decreases by 0.48%. Previous studies found a decrease in efficiency of 0.5%/1 °C^36^.

### Variable frequency drive (Inverter)

The inverter can be considered the heart of the system because of its importance. It is an electronic device that converts the direct current (DC) produced by solar panels to a suitable alternative current (AC) to operate the pump. It is also controlling the pump, regulating its work, and protecting it from changes in the current produced by solar panels. The inverter's performance was studied by studying several factors, such as its frequency, output power, and efficiency.

### Inverter frequency (Hz)

**Figure 21:**
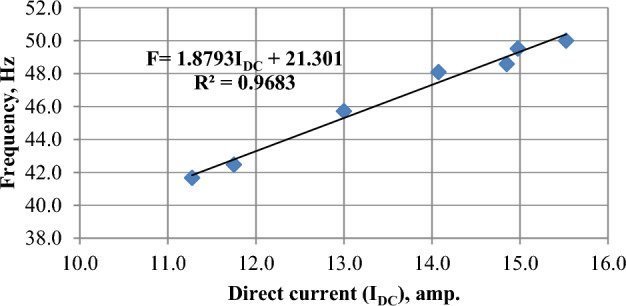
The correlation between direct current (I_DC_) and frequency (Hz).

**Figure 22:**
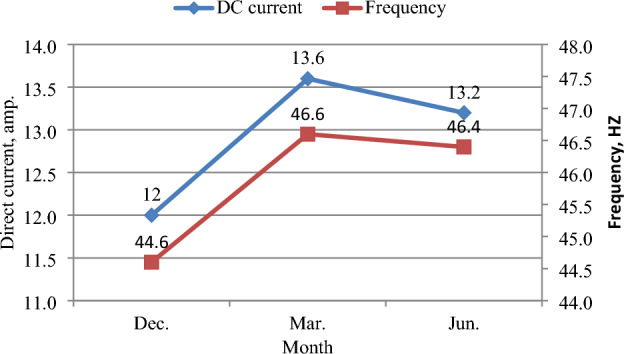
Direct current and frequency at different months.

**Figure 23:**
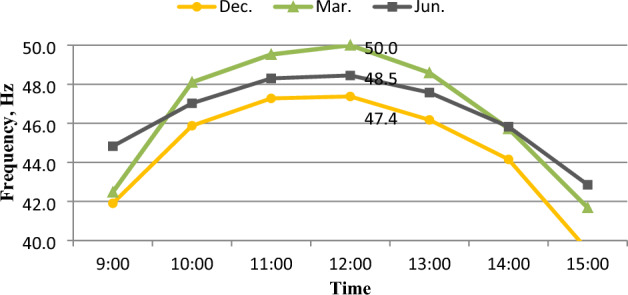
Inverter Frequency (Hz) at different months.

The inverter frequency was directly affected by the direct current produced by solar panels^37^, as shown in Fig. 21. While the highest current was in March, the average frequency reached 46.6 Hz, and the lowest current was in December, the average frequency reached 44.6 Hz. where, the average frequency value was 46.4 Hz in June as shown in Fig. 22. The highest frequency values were at 12:00 noon, that reached 47.4, 50, and 48.5 Hz in December, March, and June, respectively, as seen in Fig. 23.

### Inverter output power (AC power)

**Figure 24:**
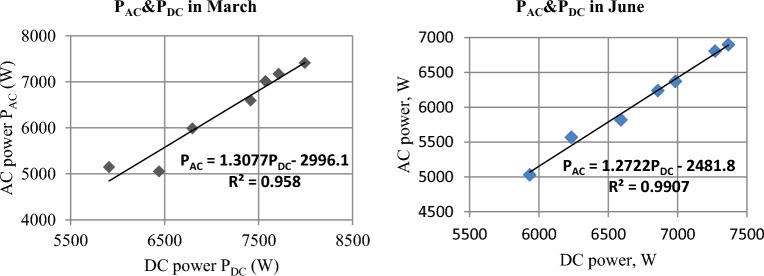
The correlation between DC Power P_DC_ and AC Power P_AC_ in March and June.

**Figure 25:**
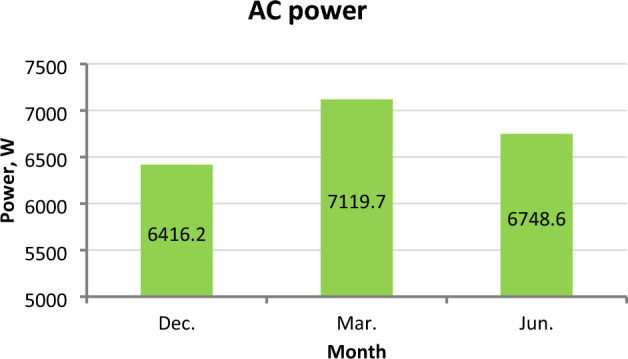
Average AC power P_AC_ at different months.

The values of electric AC power delivered by the inverter are positively dependent on the values of input electric DC power and inverter efficiency^38^. The correlation between AC and DC power is illustrated in Fig. 24 for March and June. The highest DC power value was in March, and the lowest value was in December. Therefore, it is noticeable from Fig. 25 that the highest AC power values were in March, and the lowest values were in December. Where The average AC power values reached 6416.2, 7119.7, and 6748.6 W in December, March, and June, respectively.

### Inverter efficiency

**Figure 26:**
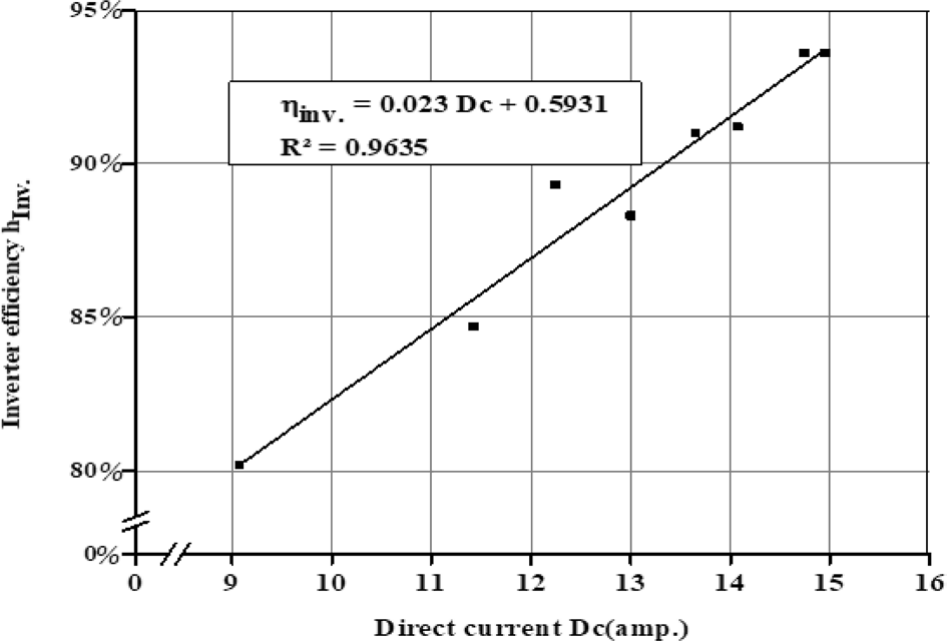
The correlation between direct current (DC) and inverter efficiency.

**Figure 27:**
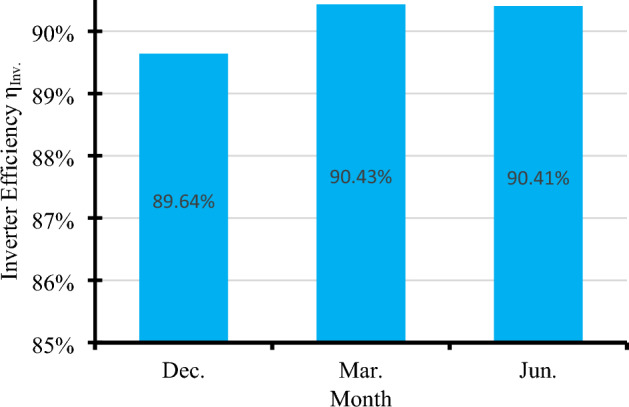
The average inverter efficiency η_inv_ in different seasons.

As illustrated in Fig. 26, direct current delivered by solar panels has a direct impact on inverter efficiency. Where the inverter should be supplied with the appropriate voltage and the appropriate direct current to operate it efficiently^38^. While the average direct current values in December and March reached 12, and 13.64, amperes, respectively. Therefore, the average inverter efficiency reached 89.64%, and 90.43%, respectively, as shown in Fig. 27.

### Pumping unit efficiency (η_pump_)

**Figure 28:**
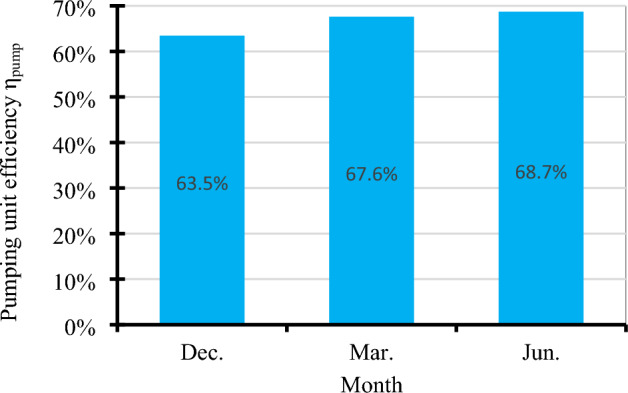
The average pumping unit efficiency at different months.

**Figure 29:**
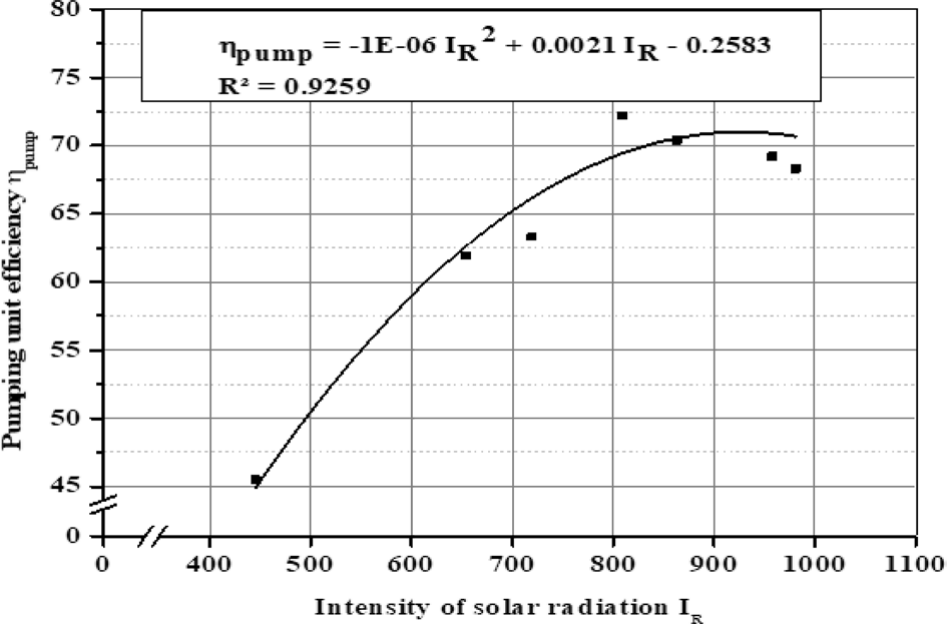
Intensity of solar radiation and pumping unit efficiency.

**Figure 30:**
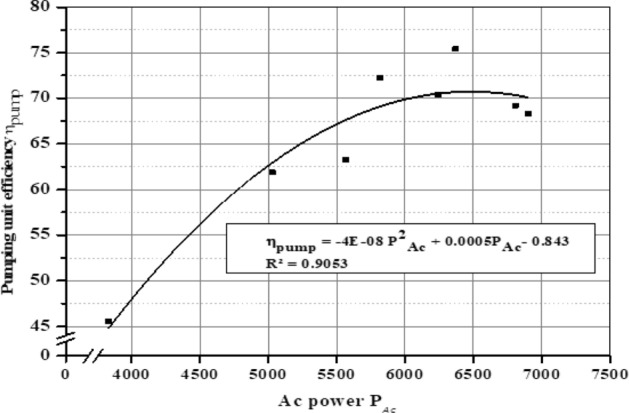
AC power and pumping unit efficiency in June.

The average pumping unit efficiency (η_pump_) for different months is shown in Fig. 28 where the efficiency (η_pump_) values in December, March, and June reached 63.5%, 67.6%, and 68.7%, respectively. It’s clear that the pumping unit efficiency (η_pump_) is affected by the intensity of solar radiation I_R_, panels temperature, and AC power (P_AC_). Figure 29 illustrate the correlation between pumping unit efficiency and solar radiation, and Fig. 30 illustrate the correlation between pumping unit efficiency AC power.

### Overall system efficiency

**Figure 31:**
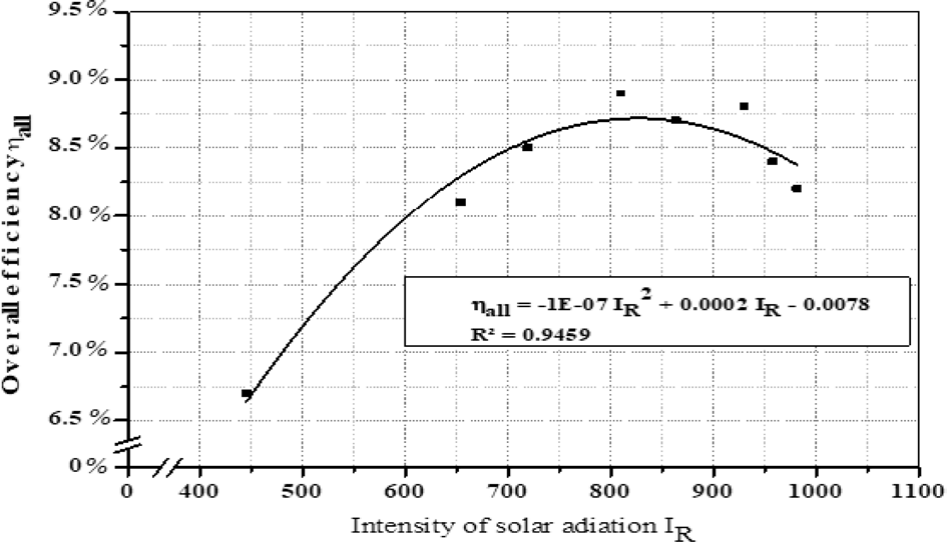
Intensity of solar radiation and overall efficiency.

The overall system efficiency can be calculated by dividing the system output (hydraulic power) by the system input (solar radiation power), or by multiplying the efficiencies of all system components, (solar panels, inverter, and pumping unit). The overall efficiency of the system is directly affected by solar radiation, but when solar radiation exceeds 900 W/m^2^ at noon, this is accompanied by an increase in temperature, which negatively affects the efficiency of the solar panels and thus the overall system efficiency, as shown in Fig. 31. In December, March, and June, respectively, the average system efficiency was 7.40%, 8.46%, and 8.51%.

## Conclusion

Solar radiation, panels' temperature, and component efficiency are the most important factors affecting the operation and performance of PV water pumping systems. The panels voltage is not significantly affected by solar radiation, where it tends to be stable, while the direct current is directly and uniformly affected by solar radiation. Furthermore, when the panel's temperature rises by 1 °C, its efficiency falls by 0.48 percent. Irradiance and the number of sunshine hours had a significant impact on the volume of water pumped during the day, which reached 129, 164.1, and 181.8 m^3^/day, respectively, in December, March, and June. In conclusion the overall average system efficiency was 7.40%, 8.46%, and 8.51%, respectively. Therefore, The results of the study showed the reliability of PV-powered underground water pumping systems, provided that negative environmental and technical factors are considered when developing and designing these systems.

## Data Availability

The datasets used and/or analyzed during the current study are available from the corresponding author on reasonable request.
